# Research considerations for prospective studies of patients with coma and disorders of consciousness

**DOI:** 10.1093/braincomms/fcae022

**Published:** 2024-01-29

**Authors:** Lorenzo Tinti, Thomas Lawson, Erika Molteni, Daniel Kondziella, Verena Rass, Tarek Sharshar, Yelena G Bodien, Joseph T Giacino, Stephan A Mayer, Moshgan Amiri, Susanne Muehlschlegel, Chethan P Venkatasubba Rao, Paul M Vespa, David K Menon, Giuseppe Citerio, Raimund Helbok, Molly McNett, The Curing Coma Campaign Collaborators, Sachin Agarwal, Venkatesh Aiyagari, Yama Akbari, Asher Albertson, Sheila Alexander, Anne Alexandrov, Ayham Alkhachroum, Fawaz Al-Mufti, Moshagan Amiri, Brian Appavu, Meron Awraris Gebrewold, Marc Ayounb, Rafael Badenes, Mary Kay Bader, Neeraj Badjiata, Ram Balu, Brooke Barlow, Megan Barra, Rachel Beekman, Ettore Beghi, Erta Beqiri, Tracey Berlin, Federico Bilotta, Thomas Bleck, Yelena Bodien, Varina Boerwinkle, Melanie Boly, Alexandra Bonnel, Luca Brazzi, Emery Brown, Sebina Bulic, Eder Caceres, Adrian Caceres, Tullio Cafiero, Elizabeth Carroll, Emilio G Cediel, Sherry Chou, Giuseppe Citerio, Jan Claassen, Chad Condie, Alfredo Conti, Katie Cosmas, Paolo Costa, Claire Creutzfeldt, Neha Dangayach, Mario Dauri, Derek Debicki, Michael DeGeorgia, Caroline Der-Nigoghossian, Masoom Desai, Rajat Dhar, Michael Diringer, Emily Durr, Brian Edlow, Ari Ercole, Anna Estraneo, Guido Falcone, Salia Farrokh, Adam Ferguson, Davinia Fernandez-Espejo, Ericka Fink, Joseph Fins, Brandon Foreman, Federico Franchi, Jennifer Frontera, Rishi Ganesan, Nicolas Gaspard, Ahmeneh Ghavam, Joseph Giacino, Christie Gibbons, Emily Gilmore, Chavie Glustein, Olivia Gosseries, Theresa Green, David Greer, Mary Guanci, Deepak Gupta, Cecil Hahn, Ryan Hakimi, Flora Hammond, Daniel F Hanley, Jed Hartings, Ahmed Hassan, Raimund Helbok, Claude Hemphill, Arthur Henrique Galvão Bruno Da Cunha, Holly Hinson, Karen Hirsch, Sarah Hocker, Peter Hu, Xiao Hu, Theresa Human, David Hwang, Judy Illes, Matthew Jaffa, Michael L James, Anna Janas, Susan Johnson, Morgan Jones, Ralf J Jox, Atul Kalanuria, Emanuela Keller, Lori Kennedy, Megan Kennelly, Maggie Keogh, Jenn Kim, Keri Kim, Hannah Kirsch, Matthew Kirschen, Nerissa Ko, Daniel Kondziella, Natalie Kreitzer, Julie Kromm, Abhay Kumar, Pedro Kurtz, Steven Laureys, Thomas Lawson, Nicolas Lejeune, Ariane Lewis, John Liang, Geoffrey Ling, Sarah Livesay, Andrea Luppi, Jennifer MacDonald, Craig Maddux, Dea Mahanes, Shraddha Mainali, Nelson Maldonado, Rennan Martins Ribeiro, Luciana Mascia, Marcello Massimini, Rohan Mathur, Stephan Mayer, Victoria McCredie, Molly McNett, Jorge Mejia-Mantilla, Michael Mendoza, David Menon, Geert Meyfroidt, Julio Mijangos, Dick Moberg, Asma Moheet, Erika Molteni, Elisa Montalenti, Martin Monti, Chris Morrison, Susanne Muehlschlegel, Marina Munar, Brooke Murtaugh, Lionel Naccache, Masao Nagayama, Emerson Nairon, Thomas Nakagawa, Andrea Naldi, Ganesalingam Narenthiran, Girija Natarajan, Esther Nemetsky, Virginia Newcombe, Niklas Nielsen, Naomi Niznick, Filipa Noronha-Falcão, Paul Nyquist, DaiWai Olson, Marwan Othman, Adrian Owen, Llewellyn Padayachy, Mehrnaz Pajoumand, Soojin Park, Melissa Pergakis, Heidi Perry, Len Polizzotto, Nader Pouratian, Marilyn Price Spivack, Lara Prisco, Javier Provencio, Francesco Puglises, Louis Puybasset, Chethan Rao, Lindsay Rasmussen, Verena Rass, Frank Rasulo, Bappaditya Ray, Zaccaria Ricci, Risa Richardson, Cassia Righy Shinotsuka, Chiara Robba, Courtney Robertson, Benjamin Rohaut, John Rolston, Stefano Romagnoli, Mario Rosanova, Eric Rosenthal, Shaun Rowe, Michael Rubin, Mary Beth Russell, Gisele Sampaio Silva, Leandro Sanz, Simone Sarasso, Aarti Sarwal, Nicolas Schiff, Caroline Schnakers, David Seder, Vishank Arun Shah, Amy Shapiro-Rosenbaubm, Angela Shapshak, Kartavya Sharma, Kumar Ajay Sharma, Tarek Sharshar, Lori Shutter, Jacobo Sitt, Beth Slomine, Keaton Smetana, Peter Smielewski, Wade Smith, Emmanuel Stamatakis, Alexis Steinberg, Robert Stevens, Jose Suarez, Gene Sung, Bethany Sussman, Shaurya Taran, Anna Teresa Mazzeo, Aurore Thibaut, David Thompson, Zachary Threlkeld, Lorenzo Tinti, Daniel Toker, Michel Torbey, Jenna Tosto, Stephen Trevick, Georgia Tsaousi, Alexis Turgeon, Andrew Udy, Panos Varelas, Paul Vespa, Walter Videtta, Henning Voss, Ford Vox, Amy Wagner, Sarah Wahlster, Mark Wainwright, John Whyte, Briana Witherspoon, Aleksandra (Sasha) Yakhkind, Susan Yeager, Michael Young, Sahar Zafar, Ross Zafonte, Darin Zahuranec, Chris Zammit, Bei Zhang, Wendy Ziai, Lara Zimmerman, Elizabeth Zink

**Affiliations:** Department of Neuroscience, Istituto di Ricerche Farmacologiche Mario Negri IRCCS, Milan 20156, Italy; Critical Care, The Ohio State University Wexner Medical Center, Columbus, OH 43210, USA; Biomedical Engineering Department, School of Biomedical Engineering and Imaging Sciences, King’s College London, London SE1 7EU, UK; Department of Neurology, Rigshospitalet, Copenhagen University Hospital, Copenhagen 2100, Denmark; Department of Clinical Medicine, University of Copenhagen, Copenhagen 2200, Denmark; Department of Neurology, Neuro-Intensive Care Unit, Medical University of Innsbruck, Innsbruck 6020, Austria; Neuro-Intensive Care Medicine, Anaesthesiology and ICU Department, GHU-Psychiatry and Neurosciences, Pole Neuro, Sainte-Anne Hospital, Institute of Psychiatry and Neurosciences of Paris, INSERM U1266, Université Paris Cité, Paris 75006, France; Department of Neurology, Harvard Medical School, Massachusetts General Hospital, Boston, MA 02114, USA; Department of Physical Medicine and Rehabilitation, Harvard Medical School, Spaulding Rehabilitation Hospital, Charlestown, MA 02129, USA; Department of Physical Medicine and Rehabilitation, Harvard Medical School, Spaulding Rehabilitation Hospital, Charlestown, MA 02129, USA; Department of Neurology, New York Medical College, Valhalla, NY 10595, USA; Department of Neurosurgery, New York Medical College, Valhalla, NY 10595, USA; Department of Neurology, Rigshospitalet, Copenhagen University Hospital, Copenhagen 2100, Denmark; Department of Neurology and Anesthesiology/Critical Care Medicine, Johns Hopkins University School of Medicine, Baltimore, MD 21205, USA; Division of Vascular Neurology and Neurocritical Care, Baylor College of Medicine and CHI Baylor St Luke’s Medical Center, Houston, TX 77030, USA; Department of Neurology, David Geffen School of Medicine at UCLA, Los Angeles, CA 90095, USA; Department of Neurosurgery, David Geffen School of Medicine at UCLA, Los Angeles, CA 90095, USA; Division of Anaesthesia, University of Cambridge, Cambridge CB2 1TN, UK; NeuroIntensive Care, IRCSS Fondazione San Gerardo dei Tintori, Monza 20900, Italy; School of Medicine and Surgery, Università Milano Bicocca, Milan 20854, Italy; Department of Neurology, Neuro-Intensive Care Unit, Medical University of Innsbruck, Innsbruck 6020, Austria; Department of Neurology, Johannes Kepler University, Linz 4040, Austria; College of Nursing, The Ohio State University, Columbus, OH 43210, USA

**Keywords:** coma, disorders of consciousness, design, outcomes, prospective studies

## Abstract

Disorders of consciousness are neurological conditions characterized by impaired arousal and awareness of self and environment. Behavioural responses are absent or are present but fluctuate. Disorders of consciousness are commonly encountered as a consequence of both acute and chronic brain injuries, yet reliable epidemiological estimates would require inclusive, operational definitions of the concept, as well as wider knowledge dissemination among involved professionals. Whereas several manifestations have been described, including coma, vegetative state/unresponsive wakefulness syndrome and minimally conscious state, a comprehensive neurobiological definition for disorders of consciousness is still lacking. The scientific literature is primarily observational, and studies-specific aetiologies lead to disorders of consciousness. Despite advances in these disease-related forms, there remains uncertainty about whether disorders of consciousness are a disease-agnostic unitary entity with a common mechanism, prognosis or treatment response paradigm. Our knowledge of disorders of consciousness has also been hampered by heterogeneity of study designs, variables, and outcomes, leading to results that are not comparable for evidence synthesis. The different backgrounds of professionals caring for patients with disorders of consciousness and the different goals at different stages of care could partly explain this variability. The Prospective Studies working group of the Neurocritical Care Society Curing Coma Campaign was established to create a platform for observational studies and future clinical trials on disorders of consciousness and coma across the continuum of care. In this narrative review, the author panel presents limitations of prior observational clinical research and outlines practical considerations for future investigations. A narrative review format was selected to ensure that the full breadth of study design considerations could be addressed and to facilitate a future consensus-based statement (e.g. via a modified Delphi) and series of recommendations. The panel convened weekly online meetings from October 2021 to December 2022. Research considerations addressed the nosographic status of disorders of consciousness, case ascertainment and verification, selection of dependent variables, choice of covariates and measurement and analysis of outcomes and covariates, aiming to promote more homogeneous designs and practices in future observational studies. The goal of this review is to inform a broad community of professionals with different backgrounds and clinical interests to address the methodological challenges imposed by the transition of care from acute to chronic stages and to streamline data gathering for patients with disorders of consciousness. A coordinated effort will be a key to allow reliable observational data synthesis and epidemiological estimates and ultimately inform condition-modifying clinical trials.

## Teaser

Tinti *et al*. outline critical considerations for prospective studies of disorders of consciousness. They emphasize the importance of selecting diverse source populations, using standardized assessment scales, and addressing challenges in case verification. The authors recommend ordinal analysis methods and comprehensive covariate measurements and provide action points to enhance research accuracy.

## Graphical Abstract

**Graphical Abstract:**
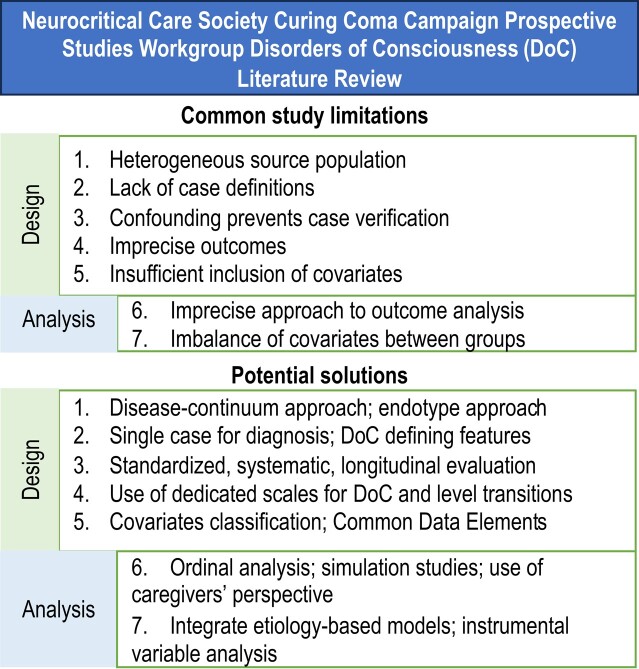

## Introduction

Acquired disorders of consciousness (DoC) encompass a spectrum of traumatic and non-traumatic brain injuries (TBIs)^1^ characterized by impaired arousal and awareness of self and environment. Behavioural responsiveness is either lacking or subtle and fluctuating. DoC precise epidemiology is still largely unknown for both acute^2^ and chronic stages.^3,4^ The scientific literature centres primarily on descriptive studies on incidence, time-based prognosis, global outcome and limited interventions for specific aetiologies leading to DoC. The field has been devoted primarily to three aetiologies of DoC, namely TBIs,^5-7^ hypoxic ischaemic encephalopathy after cardiac arrest^8^ and stroke.^9^ Uncertainty remains about whether DoC is a disease-agnostic unitary entity with a shared mechanism, prognosis or treatment response.^10^ Coordinated and systematic efforts are required to bridge knowledge among primary aetiologies. Another hurdle in DoC research is the substantial variability in managing underlying conditions, both among different aetiologies and healthcare centres.^11^ This variability increases the number of variables to account for, often leading to confounding and underpowered studies. This complexity has resulted in inconsistent inclusion criteria, varying outcomes and diverse data acquisition timings in prospective studies, hampering the comparability and generalizability of findings.^12^ These inconsistencies often arise from feasibility considerations, which depend on patient recruitment, resource availability for intervention delivery and primary and secondary endpoint assessment. Balancing feasibility, scientific rigour and clinical relevance are constant challenges when selecting variables and determining follow-up time points.

An interdisciplinary panel with experience in DoC research and care delivery participated as members of the Prospective Studies working group of the Neurocritical Care Society Curing Coma Campaign (CCC).^13^ Preliminary work within CCC has provided baseline data, which document substantial variability in DoC care across diagnoses and treatment settings.^2,14^ This manuscript provides a narrative review of observational studies of DoC, addressing common limitations and proposing strategies to promote coordinated research practices.

## Materials and methods

**Figure 1:**
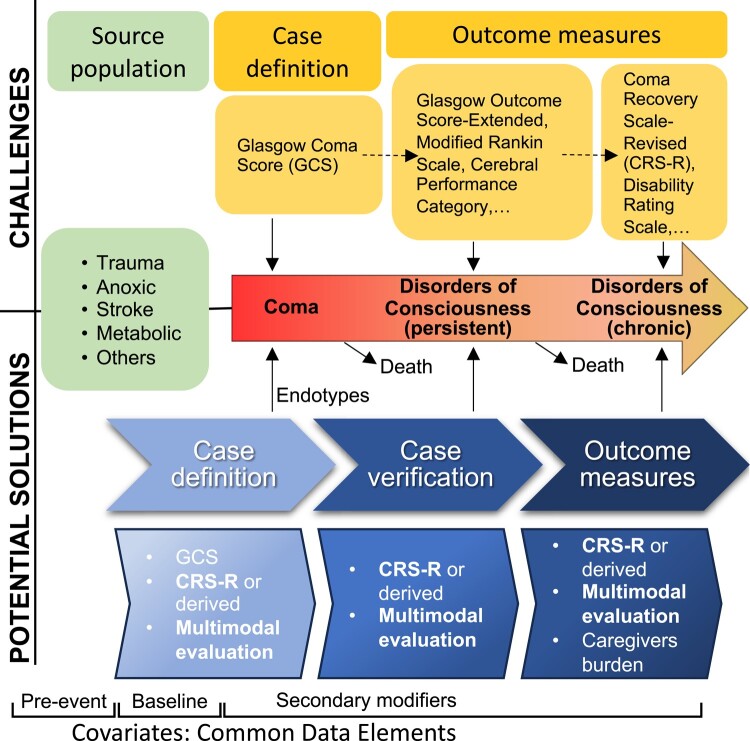
**Summary of methodological factors to be considered through the natural history of coma and disorders of consciousness.** A consensus identified issues and priority areas to be addressed for advancing coma science and disorders of consciousness care.

The primary charge of the CCC working group is to establish a platform for observational studies and future clinical trial research on DoC and coma across the continuum of care. To this aim, the working group has embarked on scientific initiatives to establish international baseline data on DoC definitions, diagnostic approaches and management practices,^12^ as well as incidence and prevalence estimates of coma across aetiologies.^2,14^ In reviewing the literature to advance these efforts, the group identified several limitations of prior prospective studies. Here, we use a narrative review approach to outline these limitations and propose mitigating strategies aimed at improving the design, feasibility, scientific rigour, reproducibility and generalizability of future prospective DoC studies (Fig. 1). These considerations were established by evidence review, synthesis and extensive panel discussion until a consensus was reached.

Data sharing is not applicable to this article as no new data were created or analysed in this study.

### Evidence review and synthesis

Two panel members performed repeated systematic literature searches between August 2021 and October 2022, with guidance from a health sciences librarian. Search terms spanned DoC levels [e.g. minimally conscious state (MCS)] and causes (e.g. stroke). Studies were included if they were original research incorporating prospective observational designs and the prognosis, treatment and/or outcomes of patients with DoC. Initial searches were supplemented with additional studies from reference libraries of the panel and recent systematic reviews.

### Panel discussion and consensus

A panel subgroup met online bi-weekly to research, discuss and synthesize evidence, reporting monthly to the larger panel. Monthly deliberations identified common themes and limitations across DoC studies, which have hindered knowledge advances and the development of clinical recommendations beyond the general definition of DoC condition. The group identified common data elements (CDEs) needed in future studies to systematically generate new knowledge across the DoC spectrum. The panel discussed the importance of pragmatism and feasibility, as well as the need to bridge research in acute and chronic phases of care and across various resource settings and cultures. Specific challenges and potential solutions for prospective DoC research are displayed in Tables 1 and 2 and described below. Delphi process was not used, as there was a need to highlight all possible limitations beyond consensus and no initial formulation of a structured Delphi thesis.

**Table 1:** Challenges and potential solutions for DoC research—design considerations

Considerations	Challenges	Potential solutions
Source population	Reliance on behavioural classification for patients with coma or DoC Composite reference population (multi-step management and care) Multiple aetiologies Multiple entry points after injury Acute stage: missing chronic follow-up Chronic stage: missing data on acute covariates	Disease continuum approach Consider multiple recruitment scenarios with flexible study entry points (reduce selection bias) Ensure homogeneous access to long-term follow-up (reduce selection bias) Retrieve acute stage covariates in studies on chronic DoC Endotype approach to DoC (e.g. ACCESS framework) to allow longitudinal investigations based on mechanistically defined subgroups Multi-modal evaluation (clinical examination, neurophysiology, neuroimaging) at subsequent steps Adopt a two-stage strategy for enrolment including caregiver evaluations Large-scale population studies including initially healthy persons
Case definition	Lack of a widely agreed, generalizable, operative definition for coma Unknown timing of the transition from coma to chronic DoC	Use of a single scale for diagnosis, capturing the defining features of DoC (e.g. CRS-R, SECONDs)
Case verification	Neurological deficits confounding clinical diagnosis (e.g. aphasia, critical illness neuropathy/myopathy) Extra-neurological impairments confounding clinical diagnosis (e.g. sepsis, medications) Cognitive motor dissociation Lack of verification of initial diagnosis	Standardized, systematic evaluation with clearly described examination protocols for clinical, neuroimaging and neurophysiology assessment Verification at successive timepoints through multi-modal tools
Selection of outcomes (dependent variables)	GOS-E, CPC and mRS provide inadequate representations of residual cognitive impairments, do not capture subtle differences in treatment effectiveness and have floor and ceiling effects Lack of precise definitions for VS/UWS or MCS in the above-mentioned scales Medical complications caused by DoC impact scores on general functioning scales	Use of dedicated scales for DoC assessment (e.g. CRS-R, SECONDs) to establish case definitions and outcome measures concurrently Define transitions between different levels of consciousness, particularly timing, trajectories and associations with medical and biological factors Development of telephone-based versions of dedicated scales to improve follow-up retention Concurrent use of traditional disability scales Integration of PROMs for patients with residual communication abilities Integration of tools for caregivers’ burden of care
Selection of covariates	Choice of relevant covariates for DoCs is not straightforward (distinction from confounders) Endpoints in previous research (e.g. in traumatic brain injury) not explicitly tailored to DoCs Small monocentric datasets for subacute and chronic DoC, inconsistently accounting for acute-phase covariates	Build conceptual frameworks to classify covariates based on different interactions with DoC constructs (e.g. pre-event, baseline and secondary modifiers) Avoid mixing processes taking place at different times Define endotype-based covariates, related to mechanisms Develop CDE for DoC to foster a standardized collection of covariates

CRS-R, Coma Recovery Scale-Revised.

**Table 2:** Challenges and potential solutions for DoC research—measurement and analytic considerations

Considerations	Challenges	Potential solutions
Study outcomes	Dichotomization of outcomes No distinction between death and chronic DoC Results may be driven by effects on mortality, prone to bias of decisions to withdraw life-sustaining therapy	Use of ordinal analysis methods, to maximize information extraction and minimize the impact of death as an extreme category in analyses Account for patients’ and caregivers’ perspectives Explore the potential of ordinal analyses to enhance statistical power in chronic DoC research Simulation studies using registry or aggregate data
Covariates	Observational data suffer from allocation bias due to the unbalance of important covariates between groups Propensity scores not explored in DoC research, possibly due to a lack of multi-variable prognostic models tailored specifically for DoC	Integrate existing aetiology-based models, models for general ICU patients and models based on previous comorbidities, into new multi-variable models for DoC Simulation studies on historical cohorts prior to the development of prospective data Instrumental variable analysis to estimate unmeasured confounding

## Summary of research considerations for prospective studies of DoC

### Design considerations

#### Source population

Patients with DoC need to be enrolled independent of hospital type and care unit, using flexible entry points to avoid selection bias and over the acute-to-chronic continuum of disease. An endotype and multi-modal approach could help with characterization and recruitment.

##### Challenges

Because DoC arises from diverse causes, selecting a reference population is crucial. However, this poses several challenges.

First, there is no established biological definition of consciousness, leading to pragmatic reliance on behavioural observations. Disagreements persist regarding the essential items for these assessments,^14,15^ and assessments are further complicated by numerous confounders in both acute and chronic stages.^16^

Secondly, DoC result from diverse clinical aetiologies, each with unique acute and chronic pathophysiological characteristics, prognostic factors and treatment approaches.

Thirdly, DoC manifestations span a continuum from coma to vegetative state/unresponsive wakefulness syndrome (VS/UWS) and MCS. However, research often fails to capture the longitudinal care of these conditions, as acute and chronic stages are typically managed by different healthcare teams with distinct treatment priorities.^14^ This leads to failure in ensuring an adequate long-term follow-up for all acute patients. Similarly, when enrolling chronic DoC patients, data on prior acute conditions are missing for certain individuals. This potentially introduces selection bias in longitudinal studies. Additionally, there is no reliable biomarker to track the neurobiological recovery process throughout the care continuum, making it challenging to assess clinical outcomes and the impact of injury mechanisms and repair processes.^17^

Finally, in longitudinal studies tracking outcomes from the time of neurologic injury, VS/UWS is a relatively uncommon outcome compared with death or other neurological disabilities.^18,19^ This rarity makes it difficult to identify predictors in the acute phase and hinders comparisons among chronic cohorts.

##### Potential solutions

Acute and chronic DoC are parts of the same disease continuum. A comprehensive approach is needed to accurately capture the incidence of acutely acquired DoC cases, independent of hospital type and care unit, using flexible entry points and ensuring homogeneous access to long-term follow-up to avoid selection bias.

The use of selected CDE^20,21^ is required to compare DoC patients across aetiologies. The use of an endotype approach^2,17^ could enable enrolment of mechanistically defined subgroups, while minimizing selection bias in longitudinal studies. The Advanced Classification of Consciousness Endotypes (ACCESS) framework can facilitate this integration.^22^ Guidelines highlight the importance of multi-modal evaluation of patients with DoC by clinical examination, EEG-based techniques and functional neuroimaging and suggest classifying their state of consciousness according to the highest level revealed by any of these three approaches.^12,23^ Guideline indications are supported by recently published case studies (e.g.^24^). In the context of prospective studies, this approach could enhance participant recruitment, by allowing identification of cases through multiple entry points along the disease continuum.

Managing variability in chronic DoC care across institutions can be challenging. Some studies have adopted a two-stage enrolment strategy,^25^ where only patients with appreciable treatment responses (by caregivers’ report) are referred for efficacy assessment. This pragmatic approach can improve recruitment in treatment trials.

Large-scale, longitudinal population studies, which are similar to the Framingham Heart Study,^26^ begin by enrolling participants prior to the DoC event. These would facilitate comprehensive tracking of the epidemiology and natural history of coma and DoC in a well-characterized cohort.^17^

#### Case definition in the acute and chronic settings

An operative definition of coma should be provided in studies until the scientific community converges to a standard. Patients’ assessment should be performed through a single, valid scale. In the intensive care unit (ICU), standard simplified scales should be routinely used.

##### Challenges

The CCC International Survey on Coma Epidemiology, Evaluation, and Therapy (COME-TOGETHER) showed marked variability in defining coma, with an overall responders’ agreement of just 64% for the expert panel’s definition.^14^ A widely agreed, generalizable, operative definition for coma is urgently needed. The Glasgow Coma Scale is widely used to grade DoC severity in the acute phase,^27^ but it lacks a universal definition for coma or other DoC.^28^

In parallel to ICU-based studies, another line of literature has evolved for subacute and chronic phases of DoC by focusing on weeks and months after the brain injury.^12^ In studies of the subacute and chronic phases of DoC, the precise timing of transition from coma to other states is generally not well documented. Relying on a 4-week threshold after injury to diagnose VS/UWS is suggested but not rooted in strong scientific evidence.^29^

##### Potential solutions

To better analyse this transition, patients enrolled in coma studies should be assessed using alternative scales as early as possible. Ideally, a single scale, like the Coma Recovery Scale-Revised (CRS-R, which was recently abbreviated, validated for ICU use, and called CRSR- for Accelerated Standardized Testing FAST),^30^ is recommended for diagnosis^12,17^ to trace natural history without gaps. The CRS-R is recommended from the ‘subacute DoC patients in the ICU, provided sedation has been stopped (or reduced as much as possible)’ to the ‘chronic patients in rehabilitation and long-term care facilities’.^23^ Its use should help early recognition of those patients whose behavioural responsiveness is limited to very subtle motor manifestations, up to cognitive motor dissociation. Detecting the appearance, presence and timing of these signs is important, as differences in prognosis might exist.^31^ In addition, the Simplified Evaluation of Consciousness Disorders (SECONDs) scale, derived from CRS-R, may allow for comparable sensitivity while reducing administration time.^32^ However, the SECONDS still awaits validation in acute DoC.

#### Case verification

Studies should identify and report confounders of DoC, establish if cognitive motor dissociation is present and leverage a multi-modal approach to verify initial diagnosis.

##### Challenges

Several factors can confound DoC diagnosis, such as inaccurate neurologic exams, incomplete syndrome assessment, sedative administration, sepsis masking the neurologic status or worsening mental status. Neurologic syndromes affecting motor or cognitive efferents may hinder behavioural expression, while sensory damage may reduce stimuli perception during exams. Notably, while routine neuroimaging can identify structural lesions, these do not always imply (invariable) disruption of the neurological function. This limitation has been acknowledged as a fundamental gap in DoC research by recent efforts by the Coma Science Work Group of the CCC.^10^ The concept of ‘cognitive motor dissociation’,^33^ defined as evidence of command following using functional MRI or EEG in the absence of behavioural evidence of conscious awareness, is gaining importance in DoC research, potentially affecting case verification in prospective study designs. Past literature has often overlooked these pitfalls.^34^ Recent research by Pincherle *et al*.^16^ retrospectively found at least one interfering factor in 75% of DoC patients evaluated early after injury (from just 48 h to within 30 days). Most frequently, signs of a possible frontal akinetic syndrome (26%), aphasia (24%), critical illness neuropathy/myopathy (12%) or non-convulsive status epilepticus (8%) were found. Across this study sample, 30% of patients had more than one complication affecting neurological examinations. Formal verification and description of initial diagnosis are also often missing in prospective DoC study designs,^4^ further jeopardizing findings.

##### Potential solutions

Future DoC studies should thus address case verification specifically, by performing serial behavioural examinations and by incorporating a standardized, systematic evaluation with clearly described imaging, neurophysiological and neurological examination processes, assessed in both the acute and chronic stages where possible. Leveraging a multi-modal approach to probing consciousness states and cognitive motor dissociation, the initial case definition should be verified at multiple timepoints throughout follow-up with either modality.^35,36^

#### Selection of outcomes (dependent variables)

Dedicated scales for the assessment of consciousness, such as CRS-R, should be used for outcome measures. These can be complemented by disability assessment scales.

##### Challenges

When DoC diagnoses are used as outcomes, researchers aim to define transitions between consciousness levels, including their timing, trajectories and associations with medical and biological factors. Describing these outcomes can be challenging, compared with more general variables like ‘community reintegration’ assessed by functioning scales.^37,38^

Different aetiologies of acute brain injury have led to the use of various, but broadly similar, scales as primary or secondary dependent. The Cerebral Performance Category (CPC) scale is commonly used for anoxic brain injury, while the modified Rankin scale (mRS) was introduced for stroke but is now recommended for other aetiologies, including anoxic brain injury.^39,40^ The Glasgow Outcome Scale-Extended (GOS-E) has been debated,^5^ and regulatory agencies have supported its use for TBI trials^41^ due to its simplicity.^42,43^ All these scales, however, have limitations: (i) they might not capture subtle differences in treatment effectiveness^44^ and have floor and ceiling effects^38,40^; moreover, dichotomizing these scales into ‘favorable’ and ‘unfavorable’ outcomes may provide an inadequate representation of residual cognitive impairments, although there have been proposals to combine these tools with cognitive tests to increase sensitivity.^41^ (ii) These scales also lack precise definitions for VS/UWS or MCS. Therefore, determining consciousness and disability levels often relies on subjective evaluations, typically by phone or mail.^38^ Subjective, unstandardized evaluations may overlook the presence of consciousness signs, because these may not test multiple interaction modalities in patients with complex neurological deficits—such as aphasia, apraxia and paresis.^45^ (iii) Patients with DoC are at increased risk for medical complications, which could impact scores on general functional scales.^46^ (iv) No research on patient-reported outcome measure (PROM) or family-reported outcome measure has been conducted in DoC to understand what outcomes matter to patients and caregivers.

##### Potential solutions

In future research, adopting a single scale like CRS-R, or the derived SECONDs, could increase the precision of DoC outcome assessment. However, phone-based versions of these tools are needed. For patients who are at the ceiling of the DoC measures, mRS, CPC and/or GOS-E could also be administered. Disability assessment can still be performed using dedicated valid instruments like Functional Independence Measure, Barthel scale or Disability Rating Scale (Fig. 1).

PROMs should be integrated into future prospective study designs, to identify what outcomes are important to patients and families.^47^ In the context of DoC, these include tools to evaluate the burden of disease on caregivers.^48,49^

Last, the majority of ICU deaths from DoC occur due to the withdrawal of life-sustaining therapies (WLSTs). However, one challenge is that many studies do not report on the mode of death (WLST versus brain death versus respiratory or cardiac arrest) or the timing of it. Patients who die due to WLST are ultimately included in the ‘unfavorable outcome’ group, despite their potential outcome being unknown. In order to understand and study mortality or adjust for potential confounding due to early WLST, studies should systematically collect and report information on the mode and timing of death. This has therefore been added in the recent CDE for DoC as a basic (mandatory) information for all studies in DoC.

#### Choice of covariates

Studies need to consider pre-event covariates, baseline covariates and secondary modifiers, to highlight causal relations and distinguish confounders and colliders.

##### Challenges

After defining populations and outcomes, specifying covariates becomes essential to identify varying trajectories or intervention responses and to assemble predictive models for individual patients. Unlike studies focused on underlying aetiologies, choosing relevant covariates for coma and DoC can be less straightforward. For example, while imaging features in TBI help predict death or severe disability,^19^ their relation to consciousness outcomes is less direct because endpoints were not explicitly tailored to DoC.

In the clinical context, we should distinguish covariates directly affecting DoC outcomes from confounders, which are variables related to both outcomes and other factors of interest. Identification of covariates is guided by both the plausibility of biological mechanisms and epidemiological correlation.^50^

Addressing the longitudinal course of these conditions is also crucial. While large datasets exist for acute-phase prognostic factors,^17^ subacute and chronic stage studies often rely on small monocentric samples and heterogeneous data gathering, prone to failure when accounting for preceding acute-phase data. Neglecting these prior events can create latent variables, increasing the heterogeneity of the natural history of recovery. On these grounds, guidelines have concluded that existing literature does not allow a reliable regression of DoC outcomes on many available covariates.^12^

##### Potential solutions

We propose a conceptual framework to classify covariates based on different interactions with DoC constructs:

1. Pre-event covariates: These are factors unrelated to injury severity, but capable of influencing the post-event trajectory. They include comorbidities, pre-event functional status or demographic characteristics. Scales such as the Charlson Comorbidity Index^51^ or Elixhauser Comorbidity Index^52^ can capture these comorbidities, while mRS^53^ or Clinical Frailty Scale^54^ stratify the premorbid functional status.
2. Baseline covariates: These covariates reflect injury severity and mainly include disease-specific data (e.g. lesion burden on neuroimaging for TBI). Aggregate scores, like the Acute Physiology and Chronic Health Evaluation-II, estimate mortality in general ICU patients based on systemic physiology,^55^ but single laboratory measures cannot discriminate contributions between pre-event damage (e.g. chronic kidney disease) and new-onset injury. Likewise, the IMPACT model considers general physiology data (glucose, haemoglobin concentration), general neurological status (pupillary reactivity) and disease-specific variables (imaging features).^19^
3. Secondary modifiers (and their biomarkers): These encompass factors like secondary damage (e.g. intracranial hypertension, seizures, hypoxia, sepsis), potential repair processes, medical complications and standard-of-care interventions, especially in the acute phase. Complications should always be considered when assessing outcomes in subacute or chronic patients, accounting for events indirectly related to the initial injury, but contributing to increased mortality and worsening prognosis.

Categorizing covariates using this framework could help unravel processes occurring at different times, aiding in highlighting causal relations between covariates and distinguishing confounders from colliders. This is essential from an epidemiological perspective to ensure appropriate statistical adjustment and prevent bias.^50^

With advancements in understanding DoC endotypes, each category will integrate covariates related to mechanisms, including parenchymal injury, structural and functional disconnections and modulatory deficits.^17^

Finally, collaborative consortia, databases or meta-analyses of the existing literature are required, as individual centres are unlikely to gather sufficiently large cohorts to be able to statistically control for all covariates. The need for standardized data collection led to the National Institute of Health—National Institute of Neurological Disorders and Stroke—launching the CDE initiative^56^ for TBI, while a similar initiative for DoC has recently been executed by the CCC.^20,21,57,58^

### Measurement and analytic considerations

#### Measurement and analysis of study outcomes

Use of ordinal scales for measurement and ordinal analysis for prediction are encouraged, to maximize information extraction and distinguish between different DoC outcomes. Results should be critically reviewed for the identification of clinically irrelevant changes, also accounting for communicating patients’ and caregivers’ perspectives.

##### Challenges

Acute-phase studies traditionally use ordinal scales and dichotomize outcomes into favourable and unfavourable categories for both treatments and prognostic factors. This approach is informative for critical care management and early family communication. However, results may be driven by effects on mortality, prone to bias of decisions to WLST. Also, dichotomization does not distinguish between death and chronic DoC, which can be questionable on several grounds:

1. ethically, it is not clear that equating death, chronic DoC and severe disability as equally unfavourable are appropriate;
2. societally, DoC entails a disability burden; and
3. scientifically, this approach hinders further DoC knowledge, since treatments target different mechanisms for survival versus DoC.

##### Potential solutions

Ordinal analysis methods, including sliding dichotomy and proportional odds analysis,^38^ have been used extensively in TBI and stroke studies to maximize the use of outcome information. Sliding dichotomy splits an ordinal scale into different points based on the baseline prognosis for individual patients. Proportional odds analysis considers all ordinal changes as meaningful outcomes (i.e. performs a ‘shift analysis’).

In conditions where prognosis may be hardly modifiable by any factors or interventions, ordinal methods can reveal subtle but meaningful changes even with small expected effect sizes. Expanding the application of ordinal analysis in DoC studies, in parallel with using dedicated ordinal scales such as CRS-R or SECONDS, might minimize the impact of death as an extreme category in analyses. One concern with ordinal analyses is the magnification of clinically insignificant improvements, irrelevant to patients’ and caregivers’ quality of life, such as transitioning from VS/UWS to severe disability on the GOS. Nonetheless, they do offer valuable insights into the overall effects of individual treatments, allowing prioritization and combination for more substantial gains in outcomes. Importantly, more research will be needed to characterize patients’ and caregivers’ conceptions of ‘significant changes’, as these might differ from medical providers’ assumptions. Future studies should also account for changes in patients’ perspectives before and after disability—a phenomenon known as ‘disability paradox’—^59,60^ by systematically implementing quality-of life-evaluations, as these may be affected differently than physical ability measures.^61,62^

In studies involving subacute and chronic patients, dichotomizing outcomes may not be suitable, given the expected limited phenotype variations. Here, the Disability Rating Scale and CRS-R offer a more accurate portrayal of trajectories. While many studies have examined effects by statistically testing median CRS-R total scores, limitations like small sample sizes or the absence of control groups often restrict outcome analyses to descriptive statistics.^25,63,64^ The potential of ordinal analyses to enhance statistical power in chronic DoC research remains unknown. Future research could benefit from simulation studies using registry or aggregate data, similar to previous efforts with acute TBI patients.^65,66^

#### Covariate measurement and analysis

Multivariable models, modelling covariates and the use of propensity scores should be considered during the study setup.

##### Challenges

Our understanding of DoC relies heavily on observational data, which makes it difficult to ascertain the individual effects of treatments or prognostic factors due to the presence of numerous confounding variables. These may lead to allocation bias due to the unbalance of important covariates between groups. Traditional strategies to mitigate this issue include selection, stratification/prognostic targeting, matching by paired analyses and adjustment using regression models.

For observational studies, propensity score analysis aims to mimic the randomization process and supports causal inference, similar to clinical trials.^67,68^ However, the use of propensity score–based methods has not been explored in DoC research, primarily because no multi-variable prognostic model tailored specifically for DoC exists. Notably, these models would also facilitate sliding dichotomy analysis for interventional trials.

##### Potential solutions

Future steps to create multi-variable models for DoC could involve integrating existing aetiology-based models,^19,69^ models for general ICU patients^55^ and models based on previous comorbidities. These typically predict mortality at various time points and thus could normalize cohorts by considering the outcome of ‘death’, ultimately enhancing DoC discrimination. As a preliminary step, simulation studies on historical cohorts could be conducted before applying these methods to prospective data.

Finally, instrumental variable analyses could help estimate unmeasured confounding in observational studies encompassing the entire DoC population,^70^ similar to simulation studies involving TBI populations and modelling causal effects.^71^

## Discussion

The CCC’s prospective working group has reviewed existing literature on prospective observational studies in DoC research and discussed challenges and opportunities for future clinical trials on DoC across the care continuum. The group identified key factors to guide future prospective research in DoC, aiming to enhance data quality and promote a coordinated approach for better science and patient care in DoC.

DoC research mainly relies on observational studies conducted in specific clinical settings. While this work offers insights into patient trajectories, there is an urgent need for robust prospective studies to delve into foundational questions about DoC biology and correlations among symptoms, states of consciousness and patient outcomes. This requires principled participant recruitment, consistent covariate selection and established measurement methods. Such a coordinated approach will facilitate the discovery of specific DoC subtypes across causes and care stages, over the longitudinal course of DoC, and beyond traditional separations among acute, post-acute, and chronic care practices.

Foundational work by the CCC substantiates these challenges and highlights the need for a coordinated and systematic approach to advance DoC science and care.^13^ The identification of priority areas underscores the need for improved endotyping of patients, biomarkers and proof-of-concept clinical trials.^72^

While available biomarkers, either imaging based or electrophysiology based, hold promise for enhancing the detection of covert consciousness states,^35,36^ their relationship with long-term prognosis in individual patients deserves further investigation.^17^ In view of this, well-designed prospective studies, through endotypic case definition and fine-grained outcomes assessment, will help link neurobiological definitions of consciousness to DoC care delivery.

This review is pragmatic, not exhaustive. While it identifies limitations in prior DoC research, it is not definitive. Our understanding of mechanisms underpinning DoC will likely evolve in the near future,^10^ prompting the scientific community to integrate new concepts about consciousness states into research protocols, adapt case definitions for patient enrolment and possibly reconsider priorities along the way. Still, prospective clinical studies on DoC will require a coordinated approach with a robust methodology to harmonize data gathering across care settings and aetiologies and to avoid bias. Further work in this direction is currently underway within the CCC and will include recommendations for harmonizing study conduct through a Delphi process among panellists of CCC working groups. The goal is to establish evidence-based practice recommendations, reduce variations in care and optimize patient outcomes.

## Data Availability

Data sharing is not applicable to this article as no new data were created or analysed in this study.

## Appendix I

Contributing collaborators of the Curing Coma Campaign are as follows: Sachin Agarwal, Venkatesh Aiyagari, Yama Akbari, Asher Albertson, Sheila Alexander, Anne Alexandrov, Ayham Alkhachroum, Fawaz Al-Mufti, Moshagan Amiri, Brian Appavu, Meron Awraris Gebrewold, Marc Ayounb, Rafael Badenes, Mary Kay Bader, Neeraj Badjiata, Ram Balu, Brooke Barlow, Megan Barra, Rachel Beekman, Ettore Beghi, Erta Beqiri, Tracey Berlin, Federico Bilotta, Thomas Bleck, Yelena Bodien, Varina Boerwinkle, Melanie Boly, Alexandra Bonnel, Luca Brazzi, Emery Brown, Sebina Bulic, Eder Caceres, Adrian Caceres, Tullio Cafiero, Elizabeth Carroll, Emilio G. Cediel, Sherry Chou, Giuseppe Citerio, Jan Claassen, Chad Condie, Alfredo Conti, Katie Cosmas, Paolo Costa, Claire Creutzfeldt, Neha Dangayach, Mario Dauri, Derek Debicki, Michael DeGeorgia, Caroline Der-Nigoghossian, Masoom Desai, Rajat Dhar, Michael Diringer, Emily Durr, Brian Edlow, Ari Ercole, Anna Estraneo, Guido Falcone, Salia Farrokh, Adam Ferguson, Davinia Fernandez-Espejo, Ericka Fink, Joseph Fins, Brandon Foreman, Federico Franchi, Jennifer Frontera, Rishi Ganesan, Nicolas Gaspard, Ahmeneh Ghavam, Joseph Giacino, Christie Gibbons, Emily Gilmore, Chavie Glustein, Olivia Gosseries, Theresa Green, David Greer, Mary Guanci, Deepak Gupta, Cecil Hahn, Ryan Hakimi, Flora Hammond, Daniel F. Hanley, Jed Hartings, Ahmed Hassan, Raimund Helbok, Claude Hemphill, Arthur Henrique Galvão Bruno Da Cunha, Holly Hinson, Karen Hirsch, Sarah Hocker, Peter Hu, Xiao Hu, Theresa Human, David Hwang, Judy Illes, Matthew Jaffa, Michael L. James, Anna Janas, Susan Johnson, Morgan Jones, Ralf J. Jox, Atul Kalanuria, Emanuela Keller, Lori Kennedy, Megan Kennelly, Maggie Keogh, Jenn Kim, Keri Kim, Hannah Kirsch, Matthew Kirschen, Nerissa Ko, Daniel Kondziella, Natalie Kreitzer, Julie Kromm, Abhay Kumar, Pedro Kurtz, Steven Laureys, Thomas Lawson, Nicolas Lejeune, Ariane Lewis, John Liang, Geoffrey Ling, Sarah Livesay, Andrea Luppi, Jennifer MacDonald, Craig Maddux, Dea Mahanes, Shraddha Mainali, Nelson Maldonado, Rennan Martins Ribeiro, Luciana Mascia, Marcello Massimini, Rohan Mathur, Stephan Mayer, Victoria McCredie, Molly McNett, Jorge Mejia-Mantilla, Michael Mendoza, David Menon, Geert Meyfroidt, Julio Mijangos, Dick Moberg, Asma Moheet, Erika Molteni, Elisa Montalenti, Martin Monti, Chris Morrison, Susanne Muehlschlegel, Marina Munar, Brooke Murtaugh, Lionel Naccache, Masao Nagayama, Emerson Nairon, Thomas Nakagawa, Andrea Naldi, Ganesalingam Narenthiran, Girija Natarajan, Esther Nemetsky, Virginia Newcombe, Niklas Nielsen, Naomi Niznick, Filipa Noronha-Falcão, Paul Nyquist, DaiWai Olson, Marwan Othman, Adrian Owen, Llewellyn Padayachy, Mehrnaz Pajoumand, Soojin Park, Melissa Pergakis, Heidi Perry, Len Polizzotto, Nader Pouratian, Marilyn Price Spivack, Lara Prisco, Javier Provencio, Francesco Puglises, Louis Puybasset, Chethan Rao, Lindsay Rasmussen, Verena Rass, Frank Rasulo, Bappaditya Ray, Zaccaria Ricci, Risa Richardson, Cassia Righy Shinotsuka, Chiara Robba, Courtney Robertson, Benjamin Rohaut, John Rolston, Stefano Romagnoli, Mario Rosanova, Eric Rosenthal, Shaun Rowe, Michael Rubin, Mary Beth Russell, Gisele Sampaio Silva, Leandro Sanz, Simone Sarasso, Aarti Sarwal, Nicolas Schiff, Caroline Schnakers, David Seder, Vishank Arun Shah, Amy Shapiro-Rosenbaubm, Angela Shapshak, Kartavya Sharma, Kumar Ajay Sharma, Tarek Sharshar, Lori Shutter, Jacobo Sitt, Beth Slomine, Keaton Smetana, Peter Smielewski, Wade Smith, Emmanuel Stamatakis, Alexis Steinberg, Robert Stevens, Jose Suarez, Gene Sung, Bethany Sussman, Shaurya Taran, Anna Teresa Mazzeo, Aurore Thibaut, David Thompson, Zachary Threlkeld, Lorenzo Tinti, Daniel Toker, Michel Torbey, Jenna Tosto, Stephen Trevick, Georgia Tsaousi, Alexis Turgeon, Andrew Udy, Panos Varelas, Paul Vespa, Walter Videtta, Henning Voss, Ford Vox, Amy Wagner, Sarah Wahlster, Mark Wainwright, John Whyte, Briana Witherspoon, Aleksandra (Sasha) Yakhkind, Susan Yeager, Michael Young, Sahar Zafar, Ross Zafonte, Darin Zahuranec, Chris Zammit, Bei Zhang, Wendy Ziai, Lara Zimmerman, and Elizabeth Zink.
